# Pain management in eldercare employees – the role of managers in addressing musculoskeletal pain and pain-related sickness absence

**DOI:** 10.1186/s12889-022-12785-x

**Published:** 2022-03-04

**Authors:** Charlotte Diana Nørregaard Rasmussen, Jodi Oakman, Kristina Karstad, Reiner Rugulies, Andreas Holtermann, Matthew Leigh Stevens

**Affiliations:** 1 National Research Centre for the Working Environment, Copenhagen, Denmark; 2 Centre for Ergonomics and Human Factors, School of Psychology and Public Health, LaTrobe University, Melbourne, Australia; 3 Department of Public Health, University of Copenhagen, Copenhagen, Denmark; 4 Department of Psychology, University of Copenhagen, Copenhagen, Denmark

## Abstract

**Purpose:**

Managers’ knowledge and behaviors in addressing musculoskeletal pain and sickness absence is not well understood. We investigated the association between managers’ knowledge and behaviours in relation to employees’ pain and their future risk of musculoskeletal pain and associated sickness absence.

**Methods:**

The prospective study included 535 eldercare employees, and 42 managers from 20 nursing homes. Managers’ self-reported knowledge and behaviors in relation to employees’ pain were grouped using Principal Components Analysis. Eldercare employees reported pain-related sickness absence, and number of days with musculoskeletal pain repeatedly over 1 year. We investigated associations using mixed-effects regression models.

**Results:**

We identified four types of managers’ knowledge and behaviors: 1) Pain-prevention (actions for prevention of employee pain), 2) Pain-management (actions to assist employees manage pain), 3) Pain-entitlements (communicating entitlements to employees with pain), and 4) Pain-accommodations (ability to facilitate workplace accommodations for employees with pain). The employees of managers with higher scores on knowledge of pain-entitlements reported fewer days of pain-related sickness absence (β = -0.62; 95%CI [-1.14; -0.10]). The employees of managers with higher scores on pain-management were more likely to report low back pain (β = 0.57; 95%CI [0.02; 1.11]). We found several key associations between the knowledge and behaviors measures and pain-related sickness absence (interactions).

**Conclusion:**

Managers’ knowledge and behaviors in relation to employees’ pain were associated with employees’ future musculoskeletal pain and sickness absence. The relationships are complex, suggesting that a multifaceted approach is needed to ensure that managers are adequately informed on how to manage and accommodate employees with musculoskeletal pain to reduce sickness absence.

**Supplementary Information:**

The online version contains supplementary material available at 10.1186/s12889-022-12785-x.

## Introduction

The recruitment and retention of employees for the eldercare sector are significant challenges across the globe and urgently require attention to manage the increased need for services due to an ageing population [1]. In Denmark, sickness absence [2, 3] and early retirement have negatively impacted [4] staffing levels in the eldercare sector, with musculoskeletal pain as a major contributing factor [2, 5]. Eldercare employees are disproportionately impacted in comparison to the general working population [2, 5–9]. This is due to the fact that among workers with pain, having manual work is a risk factor for taking sick leave [10]. However, the role of eldercare managers in assisting their employees with musculoskeletal pain to maintain their employment has not been well explored.

The prevalence of musculoskeletal pain in eldercare employees in Denmark is high, with four-weekly prevalence of low back pain (LBP) and neck-shoulder pain (NSP) reported at 61% and 72% [11].

However, despite the high levels of musculoskeletal pain, some eldercare employees are able to maintain employment [11]. For instance, Hansen and Andersen (2008) reported that more than 70% of the employees in Denmark have continued to work despite musculoskeletal pain or being ill over a 12 month period [12]. Additionally, a recent study from 2019 investigating patterns in the occurrence and duration of musculoskeletal pain and interference with work among eldercare workers, found a high one-year prevalence of pain-related work interference among eldercare employees (88%). This result shows that in spite of the experience of pain-related work interference on the majority of days, this has not been bothersome enough to take sick leave [11]. An explanation for this, could be the extensive focus in Denmark on reducing sickness absence (in particular in the public sector of eldercare work) and having appropriate accommodations to ensure good person-environment fit and maintain the ability to go to work despite of not being 100% fit/healthy.

For employees with musculoskeletal pain, having supportive management plays a central role in the maintenance of employment [12, 13], but the managers’ knowledge and competencies in the utilization of contemporary pain management strategies may be limited [11, 14–16].

Managers are in a position to create a work environment that enables employees to remain at work with musculoskeletal pain, through the communication of relevant resources and the provision of accommodations to ensure work tasks are appropriate for individuals’ capacities [15]. Organizational culture and managers who are responsive to employees’ needs have been identified as important characteristics in enabling employees to stay at work with musculoskeletal pain [17]. Managers who encourage employees to disclose their conditions and capacities for work are then in a position to facilitate appropriate working conditions, and create sustainable employment pathways for their staff despite their musculoskeletal pain [14]. Furthermore, an open relationship has been found to reduce the need for employees to take sick leave [12, 13], as they are able to discuss their condition without fear of adverse consequences [18–20]. Managers who endorse open communication are also able to fully utilize organizational policies to support their employees and optimize working conditions [16].

Many gaps remain in the knowledge of how managers can positively influence the working conditions of their employees with musculoskeletal pain and reduce sickness absence. To address these gaps, the overall aim of this study is to investigate the association of managers’ knowledge and behaviors in relation to employees’ pain and their future risk of musculoskeletal pain and pain-related sickness absence.

## Methods

We used data from the ‘Danish Observational Study of Eldercare work and musculoskeletal disorderS’ (DOSES). DOSES is a prospective workplace observational study designed to examine the associations between the physical and psychosocial working conditions, musculoskeletal pain and its consequences among eldercare employees in Danish nursing homes. For the current analysis, we used the study’s baseline and follow-up data over 1 year. A detailed description of the cohort is provided elsewhere [21].

### Study population

Study participants were 553 Danish eldercare employees, 18–65 years of age and working 15 h or more on day and evening shifts (employees only working night shifts were excluded), with a minimum of 25% of their time spent on direct resident care. Participants were recruited from 20 nursing homes and employed as care helpers (14 months of training in care provision, *n* = 262) or as care aides (additional 6 months of training, *n* = 215) [21]. For inclusion in this study, participants also had to provide baseline data on musculoskeletal pain and undertake a health check.

### Data collection

From 20 nursing homes, the team managers (*n* = 42) from every ward included in the study responded to a survey about formal and informal structures at the nursing home and wards. Eldercare employees (*n* = 553) were provided with a computer based structured questionnaire that was completed at baseline during a 45-min health check at their respective workplaces. The questionnaire included information about sociodemographics, lifestyle, health and work-related factors (for more information about the questionnaire, see the protocol paper [21]). Support was available to assist with any comprehension issues. For the current study, the questions/data used are specified in the methods section.

Managers’ knowledge and behaviours were reported by the team managers at baseline using seven items with response categories on a Likert scale ranging from 0 (strongly disagree) to 10 (strongly agree)]. The items were developed for this study and are presented in Table 1 (see also Supplementary material).

**Table 1:** Managers’ knowledge and behaviors items

Managers’ knowledge and behaviors items
“I am sure that I have enough information to help employees prevent and manage pain”
“There are things I do regularly to prevent pain among employees”
“I help clarify what options my employees have to prevent and manage pain”
“When employees have pain, I really understand how they feel”
“I am doing something active when my employees do pay attention to their pain”
“I help my employees to find out what measures they are entitled to if they have pain”
“It is easy to find solutions at work, if my employees have pain”

### Employees’ musculoskeletal pain and sickness absence

**Fig. 1:**
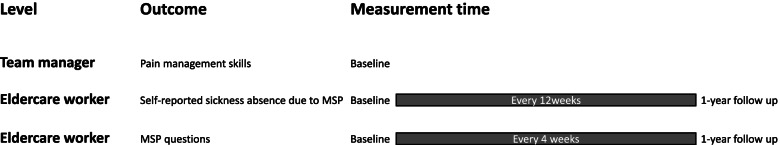
Data collection levels and time points

Outcome measures for this study were reported by the eldercare employees and collected via text messages on participants’ mobile phones (SMS delivered by the SMS Track® system (a Danish commercial system, http://www.sms-track.com/Default.aspx)). Measures included self-reported pain-related sickness absence in the previous 12 weeks (days) [22], the number of days with low back pain (LBP) in the previous four weeks and the number of days with neck/shoulder pain (NSP) in the previous four weeks measured by a slightly modified Nordic Musculoskeletal Questionnaire (NMQ) [23] (see Supplementary material). The validity and reliability of NMQ has been found to be acceptable and the NMQ has also been used extensively throughout the world and is simple to administer and well accepted by workers [24]. Sickness absence was recorded 5 times (12 weeks apart) over the 1 year follow-up period and musculoskeletal pain questions were recorded 14 times (4 weeks apart) over the same 1 year follow-up period. Figure 1 shows the data collection levels and time points.

Analyses were conducted using three further outcomes: LBP intensity (0–10), NSP intensity (0–10) [23] and total sickness absence in the previous 12 weeks (no. of days) [22]. Pain intensity scales were linked to the ‘number of days with pain’ questions. For example, if a participant reporting at least one day of LBP, they immediately received a SMS asking about their LBP intensity. Similarly for sickness absence, participants were sent an SMS asking about their total sickness absence and, if they reported at least 1 day with sickness absence, they received another SMS asking about their sickness absence due to musculoskeletal pain.

### Demographics

Demographic information was collected from participants via a baseline questionnaire. For the eldercare employees, we included sociodemographic information (i.e., age, sex), work-related characteristics (i.e. type of job and type of ward), and health and lifestyle (i.e., general health [25], LBP and NSP [23], pain-related sickness absence[22], body mass index (BMI) and smoking). For the managers, we included demographic information (age) and work-related characteristics (i.e. seniority, educational level).

### Statistical analyses

This analysis consisted of two parts. First, we analysed the questions that were asked to managers with the aim of grouping the questions into common themes/concepts that would be useful to understand in relation to worker health. We then took those concepts (or ‘factors’) and used regression modelling to determine how those concepts related to pain and sickness absence among workers.

#### Managers’ knowledge and behaviours

To identify relevant subscales from the seven items, we conducted an exploratory factor analysis, using principal components analysis with varimax rotation, using IBM SPSS Statistics for Windows, version 25.0 [26].

#### The association between managers’ knowledge and behaviours on musculoskeletal pain and sickness absence among the employees

To investigate associations between the knowledge and behaviours reported by managers on musculoskeletal pain and pain-related sickness absence among eldercare employees, we used quasi-Poisson, mixed-effects regression modelling. To select the most appropriate regression model, we first assessed the type and distribution of the data which suggested a Poisson, quasi-Poisson or negative-binomial model. The most appropriate model was selected based on QQplots of the observed versus expected residuals and Akaike information criterion (AIC) values.

Two models were constructed for each outcome. The first model included all constructs identified in the factor analysis as fixed effects. The second model included all constructs as per model 1 plus terms for the interactions between each construct. In all models, we included the nursing home, ward and individual as hierarchical levels (individuals nested within wards, wards nested within nursing homes) with random intercepts to account for any potential grouping effects of these levels. All regression analyses were conducted using R and RStudio, along with Packages tidyverse [27], glmmTMB [28], broom.mixed [29], DHARMa [30] and effects [31].

## Results

### Managers’ knowledge and behaviours

A total of 42 managers were included in this study, with a mean age of 48 years, with average experience in their current position for 4 years and 10 years of experience as a manager. Thirty-eight percent of managers had never worked as a care employee, and 38% had management training. See Table 2.

**Table 2:** Demographic and descriptive statistics for the managers

	Mean (SD), *n* (%) or median (IQR)
Age	48.9 (SD 7.2)
Time in current position (years)	4.5 (IQR 1.4 to 6.2)
Total time in management positions (years)	10.7 (IQR 6.0 to 15.0)
Time spent as a care employee	
Former	18 (43%)
Concurrent	8 (19%)
None	16 (38%)
Education level	
Qualification + additional management training	16 (38%)
Qualification as a Nurse	11 (26%)
Qualification as a Care Aide	10 (24%)
Other	5 (12%)

Data are mean or numbers

*SD* standard deviation, *IQR* interquartile range

A component structure with four factors (two with single items) was identified as optimal after examination of the scree plot and taking factor interpretability into account (see figure A in Appendix). This solution accounted for 85% of the variance in managers’ knowledge and behaviours (see table A Appendix). Factor loadings and Cronbach’s alpha are presented in table B Appendix. Constructs were named according to the major theme of the item/s:

1. **Prevention (3 items)** – actions taken by the manager for the prevention of musculoskeletal pain.
2. **Pain management (2 items)** – actions taken by the manager to assist employees with musculoskeletal pain.
3. **Entitlements (single item)** – managers’ ability to communicate on entitlements for employees with musculoskeletal pain.
4. **Workplace accommodations (single item)** – managers’ ability to find accommodations for employees with musculoskeletal pain.

### Eldercare employees’ characteristics

A total of 535 eldercare employees were included, with an average age of 45 years and mostly women (95%). Care aides (46%) or care helpers (44%) were the most common roles, in somatic wards (75%). Employees were on average slightly overweight (BMI = 26.6) and 36% were current smokers. More than 80% reported good, very good or excellent health. Median number of days recorded with LBP and NSP per month was 4. See Table 3.

**Table 3:** Demographic, work-related, health and lifestyle descriptive statistics for the eldercare employees

	Mean (SD), *n* (%) or median (IQR)
Age (years; *n* = 535)	45.3 (10.8)
Sex (female; *n* = 535)	510 (95%)
Job (*n* = 521)	
Care aide	241 (46%)
Care helper	227 (44%)
Nurse or other health professional	53 (10%)
Type of Ward (*n* = 535)	
Somatic	401 (75%)
Dementia	110 (21%)
Temporary rehabilitation	15 (3%)
Independent living	9 (2%)
BMI (*n* = 489)	26.6 (5.3)
Self-rated health (*n* = 524)	
Excellent	17 (3%)
Very good	140 (27%)
Good	282 (54%)
Not so good	78 (15%)
Poor	7 (1%)
Smoking (*n* = 525)	
Current smoker	187 (36%)
Former smoker	159 (30%)
Never smoked	179 (34%)
Days with low back pain (0–28 days; *n* = 6267*)	4 (IQR 0 to 10)
Days with neck/shoulder pain (0–28 days; *n* = 6273*)	4 (IQR 0 to 11)
Days with pain-related sickness absence (0–84 days; *n* = 2191*)	0 (IQR 0 to 0)
Responses with at least 1 day of sickness absence due to musculoskeletal pain	265 (12%)

Data are mean, numbers or median

*BMI* Body Mass Index, *SD* standard deviation, *IQR* interquartile range

*n* for these values is the number of data points (i.e., the sample population x the number of times they responded to the question)

### Managers’ knowledge and behaviours and the association with employees’ musculoskeletal pain and sickness absence

Model 1 (no interaction effects) showed no statistically significant associations between managers’ knowledge and behaviours and employees’ musculoskeletal pain (LBP or NSP) or sickness absence due to musculoskeletal pain. Coefficients ranged from -0.08 to 0.09. The marginal R^2^ (R^2^_m_) for all three outcomes (LBP, NSP and sickness absence due to musculoskeletal pain) was 0.01. Full results are available in Table 4.

**Table 4:** Multivariate analyses for the association between managers’ knowledge and behaviours and musculoskeletal pain and pain-related sickness absence among eldercare employees

	Days with LBP	Days with NSP	Sickness absence due to MSP
Model 1 (no interaction effects)			
R^2^_m_	0.01	0.01	0.01
Pain-prevention	-0.04 [-0.12; 0.05]	0.00 [-0.08; 0.08]	0.01 [-0.10; 0.12]
Pain-management	0.09 [-0.03; 0.21]	0.03 [-0.07; 0.13]	-0.08 [-0.22; 0.06]
Pain-entitlements	-0.02 [-0.10; 0.05]	-0.05 [-0.11; 0.02]	-0.02 [-0.12; 0.07]
Pain-accommodations	0.00 [-0.07; 0.07]	0.01 [-0.04; 0.06]	0.01 [-0.07; 0.09]
Model 2 (interaction effects included)			
R^2^_m_	0.02	0.01	0.09
Pain-prevention	0.12 [-0.53; 0.78]	-0.22 [-0.78; 0.34]	0.68 [-0.12; 1.47]
Pain-management	**0.57** **[0.02; 1.11]**	0.07 [-0.41; 0.56]	0.45 [-0.23; 1.13]
Pain-entitlements	0.23 [-0.18; 0.64]	0.01 [-0.34; 0.36]	**-0.62** **[-1.14; -0.10]**
Pain-accommodations	0.19 [-0.36; 0.74]	0.11 [-0.31; 0.53]	-0.19 [-0.80; 0.41]
Pain-prevention:Pain-management	-0.02 [-0.10; 0.06]	0.02 [-0.05; 0.09]	**-0.11** **[-0.21; -0.01]**
Pain-prevention:Pain-entitlements	-0.02 [-0.07; 0.03]	0.00 [-0.04; 0.04]	-0.02 [-0.08; 0.05]
Pain-prevention:Pain- accommodations	0.03 [-0.02; 0.07]	0.00 [-0.03; 0.04]	**0.06** **[0.00; 0.11]**
Pain-management: Pain-entitlements	-0.02 [-0.06; 0.03]	-0.01 [-0.05; 0.03]	0.05 [-0.01; 0.12]
Pain-management: Pain-accommodations	-0.03 [-0.10; 0.03]	-0.01 [-0.07; 0.04]	-0.06 [-0.14; 0.02]
Pain-entitlements: Pain-accommodations	-0.01 [-0.04; 0.03]	0.00 [-0.03; 0.03]	**0.05** **[0.00; 0.09]**

*LBP* Low back pain

*NSP* Neck/shoulder pain

*MSP* musculoskeletal pain

*R*^*2*^_*m*_ Marginal R^2^ (the variance explained by the fixed effects in the model)

In model 2 (including interaction effects), we identified statistically significant associations between the employees of managers who scored higher on pain management and more low back pain (β = 0.57; 95%CI = [0.02; 1.11]). Fewer days of pain-related sickness absence were reported by employees of managers who reported being able to explain entitlements than by employees whose managers reported they were not able to explain entitlements (-0.62 [-1.14; -0.10]).

We found significant interaction effects between preventive actions and pain management (-0.11 [-0.21; -0.01]), preventive actions and workplace accommodations (0.06 [0.00; 0.11]), and explanations of entitlements and workplace accommodations (0.05 [0.00; 0.09]).

**Fig. 2:**
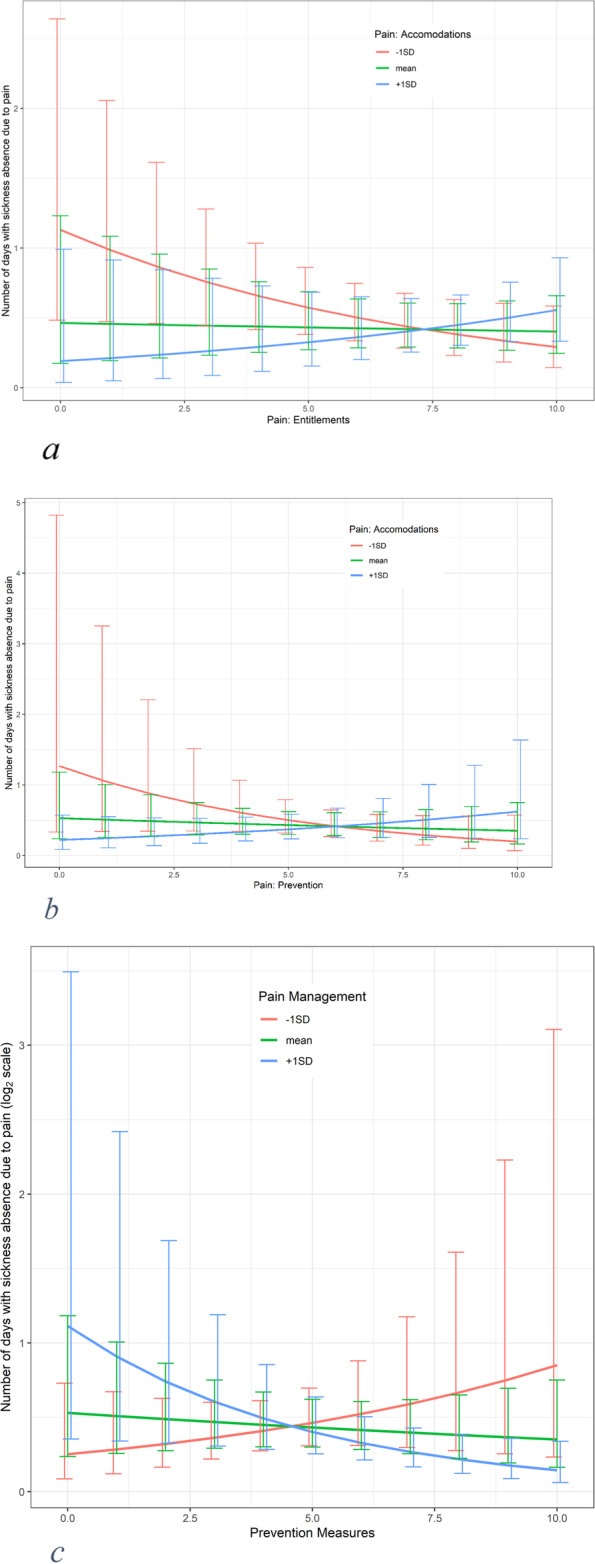
**a** Interaction between pain-entitlements and pain-accommodations on the associations with pain-related sickness absence. **b** Interaction between pain-prevention and pain-accommodations on the associations with pain-related sickness absence. **c** Interaction between pain-prevention and pain-management on the associations with pain-related sickness absence

Full results are available in Table 4 and Figs. 2a-c.

### Sensitivity analyses

In model 1 (no interaction effects) there were no statistically significant associations between managers’ knowledge and behaviours and employees’ pain intensity (LBP or NSP), or total sickness absence. For model 2 (including interaction effects) statistically significant associations were identified for total sickness absence with a statistically significant interaction effect for preventive actions times workplace accommodations (0.03 [0,00;0.06]). Full results are available in Table C in Appendix.

## Discussion

We investigated the association between managers’ knowledge and behaviors towards employees’ pain and the future risk of musculoskeletal pain and pain-related sickness absence among employees. Four types of managers’ knowledge and behaviors were identified: 1) Pain-prevention (actions for prevention of employee pain), 2) Pain-management (actions to assist employees manage their pain), 3) Pain-entitlements (communicating entitlements to assist employees with pain), and 4) Pain-accommodations (ability to facilitate workplace accommodations for employees with pain).

Employees of managers who scored higher in the area of knowledge of pain-entitlements had fewer days of pain-related sickness absence and employees of managers who scored higher on knowledge of pain-management had more low back pain. Direct relationships between the other knowledge and behaviors measures and musculoskeletal pain and pain-related sickness absence were not statistically significant. That is probably due to limited statistical power for interaction analyses. However, when we examined the influence of interactions between the different managers’ knowledge and behaviors, we found several key associations between the knowledge and behaviors measures and pain-related sickness absence. This likely reflects the complexity of pain management and the need for a nuanced and multifactorial approach to assist employees in managing their musculoskeletal pain at work. In the following sections we discuss the findings and their practical implications.

### Pain-related sickness absence

As we had expected, we found an association between managers’ knowledge and behaviors and employees’ future risk of pain-related sickness absence. The employees of managers who scored higher on knowledge of pain-entitlements had fewer days of pain-related sickness absence. This association was particularly pronounced with managers who reported lower scores on pain-accommodation compared to those with higher scores. In other words, when managers’ were not as proficient in their ability to communicate the range of entitlements available to assist employees in managing their musculoskeletal pain, or potential workplace accommodations for employees, sickness absence was higher (more days). The association between entitlements and pain-related sickness absence was also stronger when the managers reported lower knowledge of pain-prevention. Thus, ensuring that managers are well versed in the availability of entitlements for their employees and then encouraging them to communicate this information and to take preventative actions may offer opportunities for organisations to reduce absenteeism and reap economic benefits due to improved staff retention [17].

Finally, the association between managers’ pain-prevention and employee pain-related sickness absence depended on the managers skills with pain-management. Here we found that when managers reported higher knowledge of pain-management (i.e. actions taken by the manager to assist employees with musculoskeletal pain), lower scores on knowledge of pain-prevention (i.e. actions taken by the manager for the prevention of musculoskeletal pain) were associated with more sickness absence days among their employees.

These results indicate that knowledge of preventive actions for employee pain among the managers is an important factor for assisting employees with musculoskeletal pain. This is in line with findings from a previous study in eldercare workers, that found that managers’ handling of employees with pain among others depend on employee handling of—and communication about—pain, managers’ perception of their role towards employees with pain and procedures and informal approaches for handling employees with pain [14].

Overall, these findings support the need for a multifaceted approach to ensure that managers have the appropriate information to share with their employees and then the ability to develop and provide appropriate accommodations and actions to meet the capacities of employees with musculoskeletal pain.

### Musculoskeletal pain

The employees of managers who scored higher on pain-management had more low back pain.This may reflect the wording of the questions. Pain management, was covered by two questions: “When employees have pain, I really understand how they feel” and “I am doing something active when my employees have pain”. This may reflect that a proactive focus on pain from managers, will increase pain reporting among employees. Whilst the findings may appear contradictory to expectations, closer examination of the items, demonstrates their relationship to understanding of an employee’s pain and then provision of active support in staying at work. Previous research supports the need for open disclosure by employees with musculoskeletal pain to their managers [12, 13], stating that such interventions may not be effective at reducing pain levels but have other work related benefits [12]. Open disclosure is consistent with a biopsychosocial approach to pain management where minimisation of the pain is not the primary focus but enabling individuals to participate in meaningful work is important [32, 33]. The ability to discuss musculoskeletal pain conditions with a supervisor or manager enables the implementation of appropriate workplace accommodations which enable employees to stay at work and reduce associated sickness absence. Overall, the findings from the current study support previous research which has identified the importance of managers in assisting employees with musculoskeletal pain to maintain employment [14, 15, 34, 35].

### Strengths and limitations

A major strength of this study is the multilevel approach utilised for data collection and analysis. A further strength is that pain management actions of managers was not reported by the employees, but by the managers themselves. A potential limitation of this study is that (although identifying specific causal mechanisms is beyond the scope of this study) reverse causality is an issue. As such we cannot be sure whether worker health changes as a result of managers’ knowledge and behaviours, or the other way around. However, the potential impact of this issue is reduced through the prospective nature of the analysis. Another potential limitation relates to the use of self-reported sickness absence and the inability to discriminate short-term and long-term sickness absence.

### Future research

The novel and exploratory nature of our study means that replication of these findings in other studies is required. An important question is whether these findings are true for this job group only, or for other occupations, cultures and countries as well. A need exists for testing interventions for training managers in knowledge and behaviours, such as in the knowledge and then communication of entitlements and the relevant organizational processes to support those employees with musculoskeletal pain and the subsequent impact on sickness absence. Also, guidelines/tools are needed to support the managers in their roles. Finally, the knowledge and behaviours measures utilised here could be inlcluded as part of a process evaluations for interventions to support pain management by managers.

## Conclusion

Managers’ knowledge and behaviors towards employees’ pain were associated with employees’ future musculoskeletal pain and sickness absence. The complex relationships between knowledge and behaviours suggests that a multifaceted approach is needed to ensure that managers have the appropriate information to share with their employees and then have the ability to develop and provide appropriate accommodations and actions to meet the capacities of employees with musculoskeletal pain. Training of managers in contemporary pain management practices, including explanation of entitlements and the organisational process to support employees in remaining at work, may help to address the ongoing challenges with staffing in eldercare.

## Supplementary Information

**Additional file 1.**

**Additional file 2: Appendix.**

## Data Availability

The datasets generated during and analysed during the current study are not publicly available but are available from the corresponding author on reasonable request.

## Abbreviations

AIC: Akaike information criterion
BMI: Body mass index
CI: Confidence interval
DOSES: Danish Observational Study of Eldercare work and musculoskeletal disorders
IQR: Interquartile range
LBP: Low back pain
NMQ: Nordic Musculoskeletal Questionnaire
NSP: Neck shoulder pain
SD: Standard deviation
