# Supplementary material for: Pain management in eldercare employees – the role of managers in addressing musculoskeletal pain and pain-related sickness absence
Source: BMC Public Health. 2022 Mar 4;22:432. doi: 10.1186/s12889-022-12785-x (PMC8895519; doi:10.1186/s12889-022-12785-x)
Supplement: Supplementary file 1 — Additional file 1. [file 12889_2022_12785_MOESM1_ESM.docx]

Supplementary material

## Data used in the analysis.

| **Managers’ knowledge and behaviors items** |
| --- |
| “I am sure that I have enough information to help employees prevent and manage pain” |
| “There are things I do regularly to prevent pain among employees” |
| “I help clarify what options my employees have to prevent and manage pain” |
| “When employees have pain, I really understand how they feel” |
| “I am doing something active when my employees do pay attention to their pain” |
| “I help my employees to find out what measures they are entitled to if they have pain” |
| “It is easy to find solutions at work, if my employees have pain” |

Response categories: a Likert scale ranging from 0 (strongly disagree) to 10 (strongly agree).

| **Employees’ musculoskeletal pain and sickness absence** |
| --- |
| During the previous 12 weeks, how many days have you been absent from work due to illness?  [Response categories: 0-60 days] |
| During the previous four weeks, how many days have you had low back pain?  [Response categories: 0-28 days] |
| During the previous four weeks, how many days have you had neck/shoulder pain?  [Response categories: 0-28 days] |
