# Supplementary material for: Pain management in eldercare employees – the role of managers in addressing musculoskeletal pain and pain-related sickness absence
Source: BMC Public Health. 2022 Mar 4;22:432. doi: 10.1186/s12889-022-12785-x (PMC8895519; doi:10.1186/s12889-022-12785-x)
Supplement: Supplementary file 2 — Additional file 2: Appendix. [file 12889_2022_12785_MOESM2_ESM.docx]

**APPENDIX**


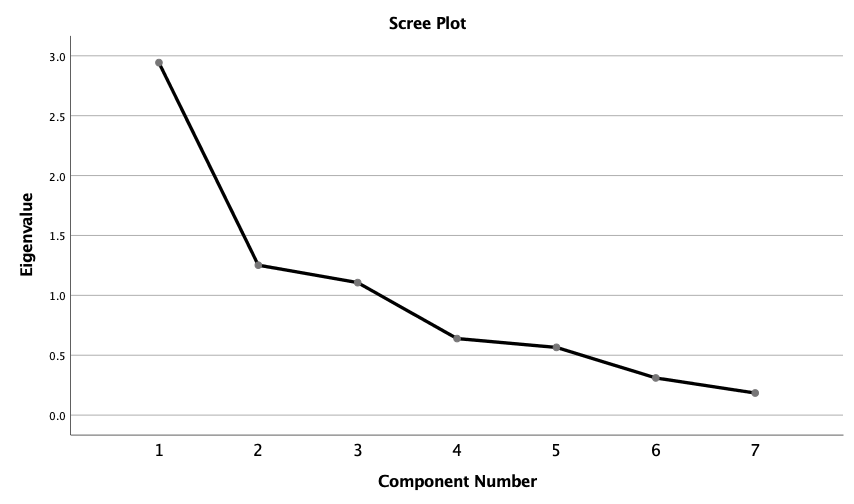


Figure A: Factor loadings

| **Total Variance Explained** | | | | | | | | | |
| --- | --- | --- | --- | --- | --- | --- | --- | --- | --- |
| Component | Initial Eigenvalues | | | Extraction Sums of Squared Loadings | | | Rotation Sums of Squared Loadings | | |
|  | Total | % of Variance | Cumulative % | Total | % of Variance | Cumulative % | Total | % of Variance | Cumulative % |
| 1 | 2.943 | 42.045 | 42.045 | 2.943 | 42.045 | 42.045 | 2.024 | 28.907 | 28.907 |
| 2 | 1.252 | 17.883 | 59.928 | 1.252 | 17.883 | 59.928 | 1.716 | 24.511 | 53.418 |
| 3 | 1.106 | 15.800 | 75.728 | 1.106 | 15.800 | 75.728 | 1.180 | 16.863 | 70.281 |
| 4 | .639 | 9.129 | 84.857 | .639 | 9.129 | 84.857 | 1.020 | 14.576 | 84.857 |
| 5 | .565 | 8.076 | 92.933 |  |  |  |  |  |  |
| 6 | .310 | 4.430 | 97.363 |  |  |  |  |  |  |
| 7 | .185 | 2.637 | 100.000 |  |  |  |  |  |  |
| Extraction Method: Principal Component Analysis. | | | | | | | | | |

Table A: Total variance explained in the factor analyses

| **Items** | **Constructs** | | | |
| --- | --- | --- | --- | --- |
|  | Prevention | Management | Entitlements | Workplace accommodation |
| I am sure that I have enough information to help employees prevent and manage pain | .85 |  |  |  |
| There are things I do regularly to prevent pain among employees | .79 |  |  |  |
| I help clarify what options my employees have to prevent and manage pain | .74 |  |  |  |
| When employees have pain, I really understand how they feel |  | .90 |  |  |
| I am doing something active when my employees have pain |  | .86 |  |  |
| I help my employees to find out what measures they are entitled to if they have pain |  |  | .92 |  |
| It is easy to find solutions at work, if my employees have pain |  |  |  | .96 |
| *Cronbach’s alpha* | .78 | .73 | Na | Na |

**Table B:** Factor loadings and cronbach’s alpha for the constructs of pain management actions

|  | LBP intensity | NSP intensity | Sickness absence (total) |
| --- | --- | --- | --- |
| Model 1 | | | |
| Est. R^2^ | 0.01 | 0.00 | 0.02 |
| Pain-prevention | -0.03  [-0.11; 0.05] | -0.01  [-0.08; 0.06] | 0.03  [-0.04; 0.10] |
| Pain-management | 0.09  [-0.02; 0.19] | 0.02  [-0.07; 0.10] | -0.05  [-0.12; 0.03] |
| Pain-entitlements | -0.02  [-0.09; 0.05] | -0.03  [-0.09; 0.03] | -0.05  [-0.11; 0.01] |
| Pain-accommodations | 0.00  [-0.06; 0.06] | 0.00  [-0.04; 0.05] | 0.02  [-0.03; 0.08] |
| Model 2 (interaction effects included) | | | |
| R^2^_m_ | 0.02 | 0.01 | 0.06 |
| Pain-prevention | 0.00  [-0.57; 0.57] | -0.26  [-0.75; 0.22] | 0.08  [-0.36; 0.51] |
| Pain-management | 0.38  [-0.10; 0.85] | -0.01  [-0.43; 0.41] | 0.22  [-0.15; 0.59] |
| Pain-entitlements | 0.25  [-0.10; 0.60] | 0.12  [-0.18; 0.42] | -0.20  [-0.47; 0.06] |
| Pain-accommodations | 0.06  [-0.42; 0.55] | 0.10  [-0.27; 0.46] | -0.01  [-0.34; 0.31] |
| Pain-prevention:Pain-management | 0.00  [-0.07; 0.07] | 0.04  [-0.02; 0.10] | -0.03  [-0.08; 0.03] |
| Pain-prevention:Pain-entitlements | -0.02  [-0.06; 0.02] | -0.01  [-0.05; 0.02] | -0.01  [-0.04; 0.02] |
| Pain-prevention: Pain- accommodations | 0.02  [-0.02; 0.06] | 0.00  [-0.03; 0.03] | **0.03**  **[0.00; 0.06]** |
| Pain-management: Pain-entitlements | -0.02  [-0.06; 0.02] | -0.01  [-0.04; 0.02] | 0.01  [-0.02; 0.04] |
| Pain-management: Pain-accommodations | -0.02  [-0.07; 0.04] | -0.01  [-0.06; 0.04] | -0.03  [-0.08; 0.01] |
| Pain-entitlements: Pain-accommodations | 0.00  [-0.03; 0.03] | 0.00  [-0.03; 0.02] | 0.02  [-0.01; 0.04] |
| LBP – Low back pain  NSP – Neck/shoulder pain  R^2^_m_ – Marginal R^2^ (the variance explained by the fixed effects in the model) | | | |

**Table C: Multivariate analyses for the association between knowledge and behaviours and pain intensity and sickness absence in the workplace.**
